# External therapy of traditional Chinese medicine for treating irritable bowel syndrome with diarrhea: A systematic review and meta-analysis

**DOI:** 10.3389/fmed.2022.940328

**Published:** 2022-08-09

**Authors:** Xiuxiu Wei, Yongtian Wen, Yuchen Wei, Xu Liang, Xiangxue Ma, Beihua Zhang, Xudong Tang

**Affiliations:** ^1^Graduate College, Beijing University of Traditional Chinese Medicine, Beijing, China; ^2^Department of Gastroenterology, Xiyuan Hospital, China Academy of Chinese Medical Sciences, Beijing, China; ^3^China Academy of Chinese Medical Sciences, Beijing, China

**Keywords:** systematic review, meta-analysis, irritable bowel syndrome with diarrhea, external therapy of TCM, randomized controlled trial, complementary therapy

## Abstract

**Background:**

Irritable bowel syndrome with diarrhea (IBS-D) is a chronic functional gastrointestinal disorder that has a significant impact on quality of life, work productivity, and healthcare resources. External therapy of traditional Chinese medicine (TCM) has positive effects on IBS-D and is simple, convenient, and low-cost. This study aimed to systematically evaluate the efficacy and safety of external therapy of TCM for IBS-D.

**Methods:**

This study was conducted according to Preferred Reporting Items for Systematic Reviews and Meta-analyses (PRISMA) guidelines. The PubMed, Embase, Cochrane Library, Web of Science, China National Knowledge Infrastructure (CNKI), Chinese Scientific Journals (VIP), Wan Fang, and Chinese Biomedical (CBM) databases were electronically searched to collect randomized controlled trials comparing external therapy of TCM with Western medicine for IBS-D from inception to 31 December 2021. Two authors independently screened, extracted, and assessed the selected studies. The Jadad scale and Cochrane Collaboration Risk of Bias tool were used to evaluate study quality. The certainty of evidence was assessed using the Grading of Recommendations, Assessment, Development, and Evaluations (GRADE). The meta-analysis was performed using the Review Manager software (version 5.3).

**Results:**

Twenty-one studies involving 1,862 subjects were included. Acupuncture and moxibustion were the most commonly used external therapies. The meta-analysis showed that based on total effective rate with moderate certainty of evidence (*n* = 21 studies, *n* = 1,862 participants, RR = 1.25, 95% CI [1.2, 1.31], I^2^ = 0%, *P* < 0.00001), clinical cure rate with low certainty of evidence (*n* = 17 studies, *n* = 1,502 participants, RR = 1.66, 95% CI [1.4, 1.96], I^2^ = 1%, *P* < 0.00001), recurrence rate with very low certainty of evidence (*n* = 5 studies, *n* = 260 participants, RR = 0.44, 95% CI [0.34, 0.58], I^2^ = 0%, *P* < 0.00001), total symptom score (MD = −4.9, 95% CI [−7.34, −2.47]), and IBS severity scoring system score (IBS-SSS) with moderate certainty of evidence (MD = −52.72, 95% CI [−63.9, −41.53]), the experimental group had significant advantages compared with the control group. The sensitivity analysis further confirmed the robustness of the primary outcomes. The improvement in quality of life associated with IBS (IBS-QOL) was superior in the experimental group compared to the control group, and the difference was statistically significant; however, the clinical heterogeneity was strong. The inverted funnel plot of the included studies indicated a potential publication bias.

**Conclusion:**

External therapy of TCM for IBS-D alleviated abdominal symptoms, improved clinical effectiveness, and reduced recurrence with great safety. However, because of the limitations of publication bias in trials, more rigorous studies with a clinical design are necessary for further verification of the outcomes.

**Systematic Review Registration:**

[https://www.crd.york.ac.uk/PROSPERO/], identifier [CRD42020222993].

## Introduction

Irritable bowel syndrome (IBS) is a chronic functional gastrointestinal disorder characterized by recurrent abdominal pain and bloating, altered bowel habits, and stool irregularities without structural or biochemical abnormalities (1). IBS is further categorized into four subtypes depending on stool consistency rather than stool frequency: IBS with constipation (IBS-C), IBS with diarrhea (IBS-D), IBS with a mixed stool pattern (IBS-M), and IBS unsubtyped (IBS-U). However, 40% of all cases are IBS-D (2, 3). Globally, the pooled prevalence of IBS is 10–20% (4). In China, the population of outpatients with IBS comprises more than half of the population attending clinics for digestive system problems, and 75% of these cases are mainly the IBS-D subtype (5). The pathophysiology of IBS is complex and poorly understood; it includes genetic predisposition, the gut-brain axis, visceral hypersensitivity, changes in the gut microbiome, alterations in gastrointestinal motility and intestinal permeability, low-grade mucosal inflammation, and immune system activation (6–10). Antidiarrheal medications, antispasmodic therapy, microecological preparations, and central neuromodulators are commonly used as medical therapies for IBS-D (11). However, their clinical efficacy for intestinal discomfort is unsatisfactory (12). Notably, IBS-D might become increasingly common because of trends in the Westernized diet and lifestyle behavior changes, thus representing a considerable burden to both healthcare service and the society because of costs of diagnosis and treatment (13, 14). IBS-D has a strong impact on the quality of life (QOL), work productivity, healthcare resources, and the society, and it has a strong economic impact because of its refractoriness and recurrence (15, 16).

The external therapy of traditional Chinese medicine (TCM) has a long history and culture. It provides a solid theoretical basis for treating various diseases and has the benefits of simplicity, convenience, and low cost. Additionally, it has been widely used for IBS-D. Several studies have shown that external therapy of TCM, such as acupuncture, moxibustion, and acupoint application, can be used to relieve symptoms and reduce the recurrence rate and adverse reactions associated with IBS-D (17–19). A variety of external therapies involving TCM has gained increasing attention because of their use as an IBS-D treatment. Furthermore, external therapy involving TCM has been included in the consensus on the diagnosis and treatment of IBS (2017 edition) for clinical guidance.

However, the clinical efficacy and safety of various external therapies involving TCM have not yet been statistically and systematically assessed. To more objectively investigate the curative effects of external therapy involving TCM for IBS-D, we collected randomized controlled trials (RCTs) of the IBS-D treatment. Then, we compared the efficacy of the external treatment of TCM with that of conventional Western medicine and conducted a meta-analysis. Additionally, we expected that our results would provide evidence-based suggestions for clinical practice and guide clinical applications more effectively.

## Materials and methods

### Study protocol

The protocol was registered with the International Prospective Register of Systematic Reviews (registration no. CRD42020222993).^1^ This study was reported according to the Preferred Reporting Items for Systematic Reviews and Meta-Analyses (PRISMA) guidelines of 2020 (20).

### Search strategy and data sources

A comprehensive search strategy for relevant clinical trials was independently performed by two reviewers (YW and YW) using the following eight databases: Web of Science, Embase, PubMed, Cochrane Library, Chinese Biomedical Database (CBM), China National Knowledge Infrastructure (CNKI), Wan Fang, and Chinese Scientific Journals (VIP). Dates ranged from the inception of each of the different databases to 31 December 2021. There were no language restrictions. Because the methods of external therapy of TCM are abundant, we conducted a literature search of the eight aforementioned databases to include the maximum number of clinical trials. Search strategies and specific details are shown in Supplementary materials.

### Study selection

#### Inclusion criteria

The following inclusion criteria for participants, intervention, comparator, study design, and study quality were used:

1) Participants: Patients diagnosed with IBS-D using specific criteria or internationally recognized criteria.
2) Intervention: The experimental group was treated with external therapy of TCM alone without any oral Chinese medicine or Western medicine.
3) Comparator: The control group was treated with conventional Western medicine treatment and without external therapy.
4) Study design: RCTs were designed with a sample size of ≥60. Moreover, treatment duration was ≥ 28 days.
5) Study quality: The methodological quality of each included study was assessed and had a Jadad score ≥3 (indicating a high-quality study) (21).

#### Exclusion criteria

The following exclusion criteria were used:

1) Duplicate publications (only the earliest publication by the same author was included).
2) Cases were not identified as the IBS-D subtype.
3) Publications were reviews, meta-analyses, animal experiments, conference abstracts, books, theses, or study protocols.
4) The control group was a self-control group or included healthy subjects without intervention.
5) Outcome measures of interest for the meta-analysis were not included.

#### Primary outcomes

The primary outcomes were total effective rate and clinical cure rate.

Criteria for total efficacy evaluation: IBS-D is a functional gastrointestinal disorder. The treatment goals for IBS-D are alleviation of abdominal pain and abdominal distension, reduction of the frequency of defecation, and improvement in stool form; the achievement of these goals was based on the participants’ self-assessment of diarrhea symptoms. Clinical effect was assessed based on changes in the patients’ self-reported major symptom scores. Total effectiveness rate was assessed using the comprehensive symptom score index (CSSI). The formula for calculating the CSSI was as follows: CSSI (%) = (score before−score after)/score before × 100%. When the CSSI (%) was ≥30%, a clinical effect was considered. If the CSSI (%) reached 90% or higher, then clinical recovery was considered. Treatments were recorded as effective, markedly effective, and clinically curative.

#### Secondary outcomes

Secondary outcomes were recurrence rate, total symptom score, IBS severity scoring system (IBS-SSS) questionnaire score, and IBS-QOL scale score.

### Research screening process

First, duplicate records were eliminated according to the parameters of the title and author information using the NoteExpress software (Beijing Aegean Sea Music Technology Co., Ltd., Beijing, China). Subsequently, the remaining abstracts and full texts were independently reviewed by two authors using the inclusion and exclusion criteria (YW and YW). Disagreements were resolved by negotiation.

### Data extraction and management

The two authors (W and YW) separately extracted the following data from each included study using predesigned tables: general information such as title, first author, and year of publication, study features such as method of randomization, allocation sequence generation, assignment concealment, blinding, and withdrawal, and data details such as sample size, age, disease duration, diagnostic criteria, outcome measures, intervention, duration, and adverse events. The included clinical trials were scored using the Jadad scale (22). Discrepancies between the two authors were resolved by a discussion resulting in a consensus, or an assessment by a third reviewer (XM).

### Quality assessment

The methodological quality of the trials was assessed independently by two reviewers using the Jadad scale and the Cochrane Collaboration Risk of Bias tool. The quality of the studies used during this review was evaluated using the Jadad scale, which included randomization, blinding, and withdrawals and dropouts (22). Moreover, the risk of bias of the RCTs was appraised using the Cochrane Collaboration tool, which is composed of the following seven domains: random sequence generation, allocation concealment, blinding of participants and personnel, blinding of the outcome assessment, incomplete outcome data, selective reporting, and other sources of bias. Three levels of bias, high risk, low risk, and unclear risk, were used to assess each domain, and graphs depicting this information were visualized. A funnel plot was used to assess for publication bias when the pooled analyses included more than 10 studies.

### Grading certainty of evidence

The quality of evidence assessment of the primary and secondary outcomes was determined using Grading of Recommendations, Assessment, Development, and Evaluation (GRADE) (23).

### Statistical analysis

Data synthesis and statistical analysis using a random effects model were performed using Review Manager (version 5.3). For dichotomous outcomes, the relative risk (RR) and 95% confidence intervals (CIs) were presented as effects measured using the Mantel-Haenszel method. For continuous variable outcomes, weighted mean differences (MDs) and 95% CIs were presented as effect measures. Forest plots were used to display summary statistics. I^2^ statistics and the chi-square test method were used to statistically evaluate the heterogeneity of the included studies. I^2^ statistics < 50% and *p* > 0.1 indicated no significant heterogeneity across the studies. I^2^ statistics > 50% and *p* < 0.1 indicated significant heterogeneity across the studies. When the results of the I^2^ statistics and *p*-values were inconsistent, I^2^ statistics evaluation was selected as the main assessment method. Furthermore, a sensitivity analysis was performed to evaluate the robustness of the primary outcomes. Additionally, subgroup analyses were conducted based on the types of external therapy of TCM to explore whether the results had changed.

## Results

### Search results and study characteristics

**FIGURE 1:**
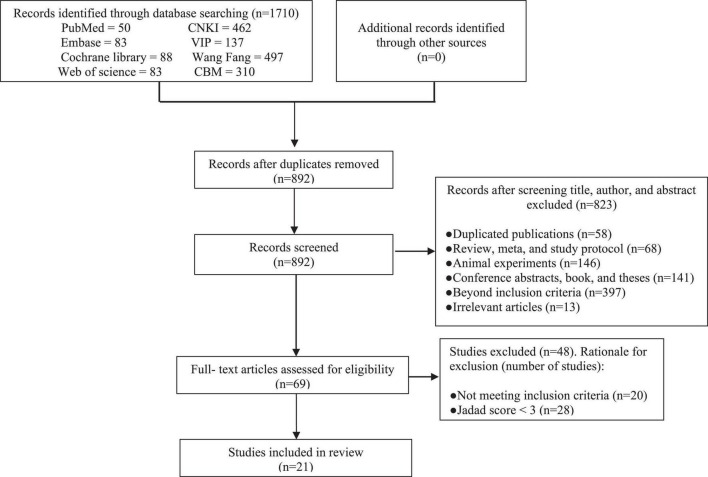
Flow chart of study selection process.

A total of 1,710 relevant RCTs were initially retrieved. After gradual screening, we identified 21 studies involving 1,862 subjects, including 983 in the experimental group and 879 in the control group (Figure 1). The subjects were 17–71 years of age, and disease duration ranged from 3 months to 20 years. The maximum sample size of the included studies was 231. There were 13 studies on acupuncture therapy, including acupuncture, electroacupuncture, eye acupuncture, and head acupuncture (24–36). Five studies on moxibustion therapy included herb-separated moxibustion, long-snake moxibustion, warming acupuncture moxibustion, and umbilical moxibustion (37–41). Three studies on acupuncture included acupoint application (42–44). Acupuncture and moxibustion therapy are the common external treatment methods used in most of the studies. The treatment methods used for the control group were mainly antispasmodic, antidiarrheal, or adjusted intestinal flora. In terms of treatment duration, most of the studies performed treatment for 4 weeks, and only one study performed treatment for up to 16 weeks (30). Only five studies recorded follow-up durations of 3 or 6 months (25, 26, 40, 41, 43). The basic characteristics of the included trials are detailed in Table 1.

**TABLE 1:** Basic characteristics of the included studies.

Study	Sample size(n) (E/C)	Age (E/C)	Disease course (E/C)	Diagnostic criteria	Diagnostic criteria of traditional Chinese medicine	Syndrome types of traditional Chinese medicine	Evaluation Criteria for outcomes	Interventions		Treatment durtion	Follow-up duration	Adverse event		Jadad	Outcomes
								E	C			E	C		
Liu et al. (24)	31/32	38.15 ± 7.85/38.45 ± 8.22	26.22 ± 7.08m/25.88 ± 7.02m	Rome III	Guidelines of Clinical Research of New Drugs of Traditional Chinese Medicine (Trial)	Gan- stagnancy Pi-deficiency syndrome	Guidelines of Clinical Research of New Drugs of Traditional Chinese Medicine (Trial)	Acupuncture; 40 sessions over 8 weeks	Trimebutine maleate capsules (0.2g, 3 × /day, 5days/week)	8 weeks	NA	NA	NA	3	①②④⑤
Mao (25)	40/40	46.38 ± 11.47/47.49 ± 12.39	96.49 ± 45.54m/90.13 ± 47.93m	Rome III	Not mentioned	Not mentioned	Guidelines of Clinical Research of New Drugs of Traditional Chinese Medicine (Trial)	Acupuncture; 18 sessions over 6 weeks	Pinaverium bromide (50mg, 3 × /day)	6 weeks	18 weeks	1 case with anxiety and depression, 1 case with mild blood sickness	4 cases with mild nausea and vomiting	3	①②⑤⑥
Li et al. (26)	35/35	39.1 ± 11.8/37.9 ± 11.5	4.33 ± 3.93y/5.23 ± 7.35y	Rome II	Consensus on TCM Diagnosis and Treatment of Irritable Bowel Syndrome	Gan- stagnancy Pi-deficiency syndrome	Improvement of clinical symptoms	Acupuncture; 12∼16 sessions over 4 weeks	Pinaverium bromide (50mg, 3 × /day)	4 weeks	3 months	0	0	3	①②③④⑥
Lu (27)	38/38	54.59 ± 12.50/54.54 ± 11.96	5.86 ± 3.44y/5.86 ± 3.68y	Rome III	Consensus on TCM Diagnosis and Treatment of Irritable Bowel Syndrome and Guidelines for the Diagnosis and Treatment of Digestive Diseases in Traditional Chinese Medicine (2006 Edition)	Gan- stagnancy Pi-deficiency syndrome	Improvement of clinical symptoms	Acupuncture; 12 sessions over 4 weeks	Pinaverium bromide (50mg, 3 × /day)	4 weeks	NA	NA	NA	3	①②④
Sun et al. (28)	30/30	38.81 ± 11.80/38.59 ± 11.45	4.23 ± 3.96y/5.63 ± 7.35y	Rome III	Guidelines of Clinical Research of New Drugs of Traditional Chinese Medicine	Gan- stagnancy Pi-deficiency syndrome	Guidelines of Clinical Research of New Drugs of Traditional Chinese Medicine	Acupuncture; 20 sessions over 4 weeks	Pinaverium bromide (50mg, 3 × /day)	4 weeks	NA	0	0	3	①②④⑥
Zhang et al. (29)	31/30	39.5 ± 2.1/39.9 ± 2.1	71.0 ± 8.4m/69.4 ± 7.6m	Rome III	Not mentioned	Not mentioned	Improvement of IBS-SSS score	Acupuncture; 12 sessions over 4 weeks	Pinaverium bromide (50mg, 3 × /day)	4 weeks	NA	NA	NA	3	①②⑤
Cao et al. (30)	35/35	44.36 ± 8.61/44.05 ± 8.72	8.52 ± 5.03y/8.84 ± 5.25y	Rome III	Guidelines of Clinical Research of New Drugs of Traditional Chinese Medicine	Gan- stagnancy Pi-deficiency syndrome	Guidelines of Clinical Research of New Drugs of Traditional Chinese Medicineand and the Standard of Cure and Improvement of Clinical Diseases	Acupuncture; 80 sessions over 16 weeks	Pinaverium bromide (50mg, 3 × /day)	16 weeks	NA	NA	NA	3	①②⑤
Li et al. (31)	51/26	46 ± 13/48 ± 13	143.6 ± 125.9m/133.3 ± 116.7m	Rome III	Not mentioned	Not mentioned	Improvement of IBS-SSS score	Acupuncture; 18 sessions over 6 weeks	Pinaverium bromide (50mg, 3 × /day)	6 weeks	NA	0	1 patient had severe diarrhea after taking pinaverium bromide, and withdrew.	3	①②
Li et al. (32)	30/30	46 ± 16/44 ± 16	13.6 ± 9.8y/13.3 ± 10.1y	Roma III	Guidelines of Clinical Research of New Drugs of Traditional Chinese Medicine (Trial)	Gan- stagnancy Pi-deficiency syndrome	Guidelines of Clinical Research of New Drugs of Traditional Chinese Medicine	Acupuncture; 28 sessions over 8 weeks	Pinaverium bromide (50mg, 3 × /day)	8 weeks	NA	NA	NA	3	①
Guo et al. (33)	154/77	46 ± 12/44 ± 13	6∼480m/6∼348m	Rome III	Not mentioned	Not mentioned	Improvement of IBS-SSS score	Acupuncture; 18 sessions over 6 weeks	Pinaverium bromide (50mg, 3 × /day)	6 weeks	NA	7 cases with subcutaneous hemorrhage	2 cases with dry mouth, 2 cases with dizziness, and 1 case with nausea	3	①②⑤⑥
Shi et al. (34)	60/60	40.2 ± 10.8/38.5 ± 9.1	8.6 ± 3.8y/7.3 ± 2.1y	Rome III	Not mentioned	Not mentioned	Guidelines of Clinical Research of New Drugs of Traditional Chinese Medicine	Electro acupuncture	Trimebutine maleate capsules (0.2g, 3 × /day)	8 weeks	NA	NA	NA	3	①
Wan et al. (35)	58/57	37.23 ± 10.21/40.07 ± 11.67	4.04 ± 1.13y/4.12 ± 1.78y	Rome III	Diagnosis and Treatment of Irritable Bowel Syndrome with Integrated Chinese and Western Medicine	Gan- stagnancy Pi-deficiency syndrome, Deficiency of both Spleen and stomach syndrome	Guidelines of Clinical Research of New Drugs of Traditional Chinese Medicine	Eye-acupuncture; 28 sessions over 4 weeks	Pinaverium bromide (50mg, 3 × /day)	4 weeks	NA	NA	NA	3	①②③④
Zhang et al. (36)	50/50	21∼71/23∼68	3m∼15y/8m∼20y	Rome III	Guidelines of Clinical Research of New Drugs of Traditional Chinese Medicine	Gan- stagnancy Pi-deficiency syndrome	Guidelines of Clinical Research of New Drugs of Traditional Chinese Medicine	Head-acupuncture; 30 sessions over 30 days	Bifidobacterium capsule (2 capsules, 2 × /day)	30 days	NA	NA	NA	3	①②
Liu et al. (37)	38/37	37.05 ± 7.88/38.01 ± 8.01	25.39 ± 6.25m/24.97 ± 7.79m	Rome IV	Guidelines of Clinical Research of New Drugs of Traditional Chinese Medicine (Trial)	Gan- stagnancy Pi-deficiency syndrome	Guidelines of Clinical Research of New Drugs of Traditional Chinese Medicine (Trial)	Herb-separated moxibustion; 40 sessions over 8 weeks	Pinaverium bromide (50mg, 3 × /day)	8 weeks	NA	NA	NA	3	①②④⑤
Hao and Shi (38)	42/42	38 ± 8/37 ± 7	23 ± 7m/24 ± 8m	Rome III	Guidelines of Clinical Research of New Drugs of Traditional Chinese Medicine (Trial)	Gan- stagnancy Pi-deficiency syndrome	Guidelines of Clinical Research of New Drugs of Traditional Chinese Medicine (Trial)	Herb-separated moxibustion; 40 sessions over 8 weeks	Pinaverium bromide (50mg, 3 × /day)	8 weeks	NA	NA	NA	3	①②④⑤
Geng et al. (39)	30/30	35.98 ± 7.39/35.29 ± 7.12	3.45 ± 1.03y/3.12 ± 0.98y	Rome III	Not mentioned	Spleen and kidney yang deficiency	Improvement of clinical symptoms	Long-snake moxibustion; 8 sessions over 8 weeks	Loperamide hydrochloride capsule(2mg, 3 × /day) + Bacillus licheniformis live capsule(0.5g, 3 × /day)	8 weeks	NA	NA	NA	3	①②④⑤
Ge and Zeng (40)	60/60	38.9 ± 11.2/39.1 ± 10.3	1∼13y/1∼12y	Rome II	Not mentioned	Not mentioned	Improvement of clinical symptoms	Warming acupuncture-moxibustion; 24 sessions over 4 weeks	Loperamide hydrochloride capsules (2mg, 3 × /day)	4 weeks	6 months	NA	NA	3	①②③
Li et al. (41)	30/30	44.27 ± 11.95/44.17 ± 13.78	6.77 ± 2.93y/7.27 ± 3.04y	Rome III	Consensus on TCM Diagnosis and Treatment of Irritable Bowel Syndrome	Gan- stagnancy Pi-deficiency syndrome	Guidelines of Clinical Research of New Drugs of Traditional Chinese Medicine (Trial)	Umbilical moxibustion; 20 sessions over 4 weeks	Pinaverium bromide (50mg, 3 × /day)	4 weeks	3 months	NA	NA	3	①②③
Gu (42)	30/30	38.24 ± 11.32/37.53 ± 10.21	5.63 ± 1.22y/6.55 ± 1.54y	Rome III	Guidelines of Clinical Research of New Drugs of Traditional Chinese Medicine (Trial)	Gan- stagnancy Pi-deficiency syndrome	Guidelines of Clinical Research of New Drugs of Traditional Chinese Medicine	Acupuncture and acupoint application; 20 sessions over 4 weeks	Pinaverium bromide (50mg, 3 × /day)	4 weeks	NA	NA	NA	3	①④
Luo et al. (43)	50/50	42.3 ± 9.8/40.5 ± 10.1	3.4 ± 1.5y/3.7 ± 1.0y	Rome III	Guidelines of Clinical Research of New Drugs of Traditional Chinese Medicine (Trial)	Not mentioned	Consensus on TCM Diagnosis and Treatment of Irritable Bowel Syndrome	Acupuncture and acupoint application; 14 sessions over 4 weeks	Trimebutine maleate capsules (0.2g, 3 × /day)	4 weeks	6 months	0	0	3	①②③④⑥
Lin et al. (44)	60/60	40.2 ± 10.8/38.5 ± 9.1	8.6 ± 3.8y/7.3 ± 2.1y	Rome III	Guidelines of Clinical Research of New Drugs of Traditional Chinese Medicine (Trial)	Not mentioned	Guidelines of Clinical Research of New Drugs of Traditional Chinese Medicine	Acupuncture and acupoint application	Trimebutine maleate capsules (0.2g, 3 × /day)	4 weeks	NA	NA	NA	3	①

E, experimental group; C, control group; m, month; y, year. ① total effectiveness rate; ② clinical cure rate; ③ recurrence rate; ④ total symptom score; ⑤ score on irritable bowel syndrome-severity scoring system score questionnaire; ⑥ score on Irritable bowel syndrome-quality of life scale.

### Risk of bias and methodological quality assessment

**FIGURE 2:**
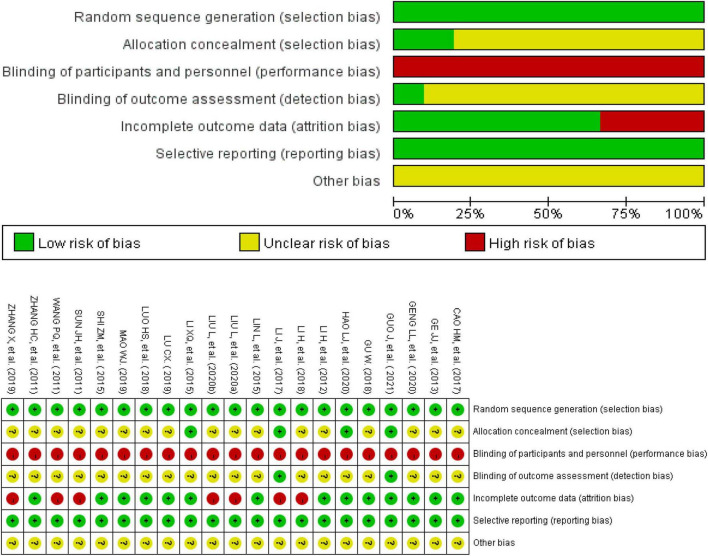
Risk of bias graph and summary. red, high risk; green, low risk; yellow, unclear risk.

All the trials reported appropriate random sequence generation methods and were rated as low risk. Four studies recorded adequate information about the methods used for allocation concealment and were rated as low risk (31–33, 38). The allocation concealment of the others was not mentioned and rated as unclear. Because of the particularity of the intervention methods, the participants and personnel were not blinded. Therefore, the performance bias of all the trials was rated as high risk. Two trials described the method of blinding of assessors and were rated as low risk (31, 33). Seven studies mentioned that the participants withdrew or dropped out of the trials, and they included abscission data that were not included in the analysis; these were rated as high risk (24, 28, 29, 33, 35, 37, 41). The remaining studies with all data included in the final analysis were rated as low risk. Additionally, it could not be judged whether there was other bias in the 21 studies; therefore, they were rated as unclear. The results of the risk of bias analysis in the included trials are summarized in Figure 2.

### Primary outcomes

#### Total effectiveness rate

**FIGURE 3:**
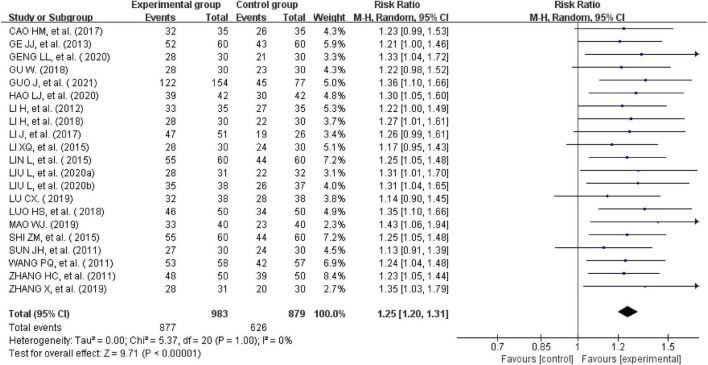
Forest plot of the total effectiveness rate.

**FIGURE 4:**
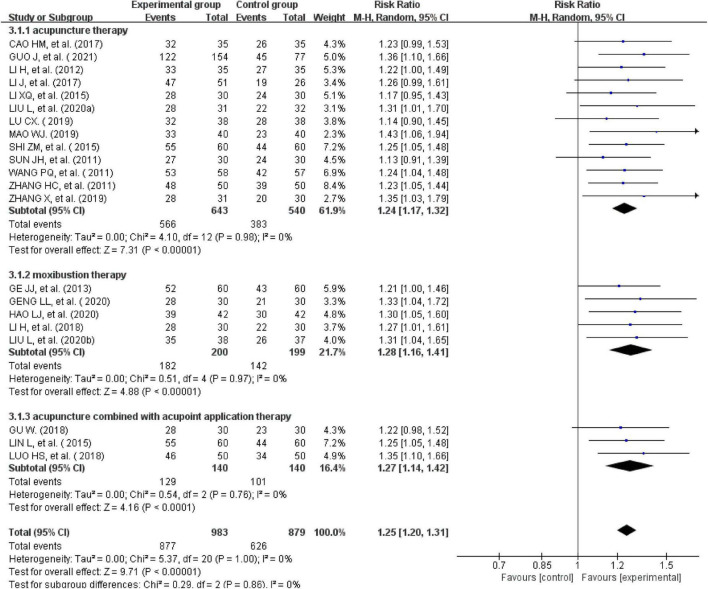
Forest plot of the effective rate of subgroups.

All the 21studies compared the total effectiveness rates of the experimental and control groups. Effectiveness was measured using the scores of the main symptoms. Twelve studies with a CSSI ≥ 30%were evaluated as effective (24, 26, 28, 31, 34–39, 41, 44). One study with a CSSI ≥ 35% was evaluated as effective (27). Additionally, four studies (25, 29, 31, 33) were evaluated using the IBS-SSS score, and four trials (20, 30, 32, 37) were evaluated based on patients’ self-reported scores for symptoms such as abdominal pain, abdominal distension, frequency of defecation, and stool form (30, 40, 42, 43). There was no heterogeneity across the trials when tested using I^2^statistics (df = 20, I^2^ = 0%). The meta-analysis showed that external therapy with TCM had a significantly higher total effectiveness rate in the experimental group than in the control group, and that the difference was statistically significant (*n* = 1,862, RR = 1.25, 95% CI [1.2, 1.31], *Z* = 9.71, *P* < 0.00001; Figure 3). The 21 included studies were further removed individually for the sensitivity analysis, which showed that none of the studies significantly affected the results of this analysis, indicating that it had great reliability and stability. This showed that the total clinical effectiveness rate of external therapy with TCM alone for IBS-D was better than that of the control group treated with internal Western medicine. Moreover, a subgroup analysis was conducted after various external treatments. The subgroup analysis showed that compared with the control group, acupuncture therapy (*n* = 1,183, I^2^ = 0%, RR = 1.24, 95% CI [1.17, 1.32], *Z* = 7.31, *P* < 0.00001), moxibustion therapy (*n* = 399, I^2^ = 0%, RR = 1.28, 95% CI [1.16, 1.41], *Z* = 4.88, *P* < 0.00001), and acupuncture combined with acupoint application therapy (*n* = 280, I^2^ = 0%, RR = 1.27, 95% CI [1.14, 1.42], *Z* = 4.16, *P* < 0.00001) had greater total effectiveness rates, indicating that the difference was statistically significant (Figure 4).

#### Clinical cure rate

**FIGURE 5:**
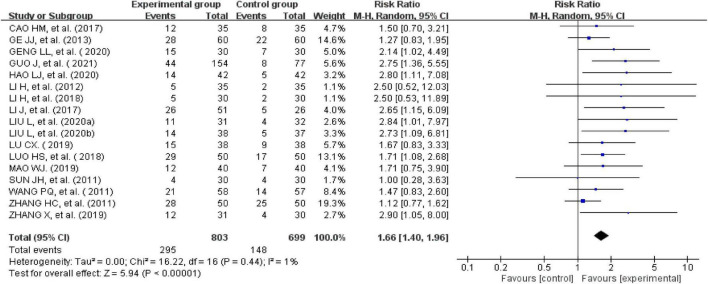
Forest plot of the clinical cure rate.

The clinical cure rate was calculated as follows: number of cured cases/total number × 100%. Seventeen studies reported clinical cure rates (24–31, 33, 35–41, 43). There was no significant heterogeneity across the included studies when tested using I^2^statistics (df = 16; I^2^ = 1%). The meta-analysis showed that the experimental group receiving external therapy of TCM had a significantly higher clinical cure rate than the control group; this difference was statistically significant (*n* = 1,502, RR = 1.66, 95% CI [1.4, 1.96], *Z* = 5.94, *P* < 0.00001; Figure 5). The sensitivity analysis showed that none of the studies significantly interfered with the results of the analysis, indicating that this study had satisfactory reliability and stability.

### Secondary outcomes

#### Recurrence rate

**FIGURE 6:**
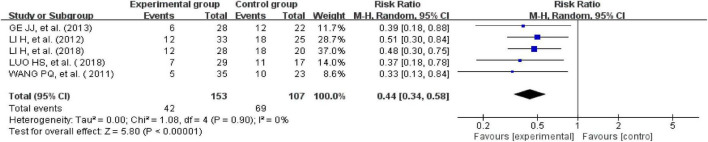
Forest plot of the recurrence rate.

Five studies reported recurrence rates (26, 35, 40, 41, 43). There was no heterogeneity across the included trials when tested using I^2^ statistics (df = 4; I^2^ = 0%). The meta-analysis showed that the experimental group receiving external therapy of TCM had a significantly lower clinical cure rate than the control group; this difference was statistically significant (*n* = 260, RR = 0.44, 95% CI [0.34, 0.58], *Z* = 5.8, *P* < 0.00001; Figure 6).

#### Total symptom score

**FIGURE 7:**
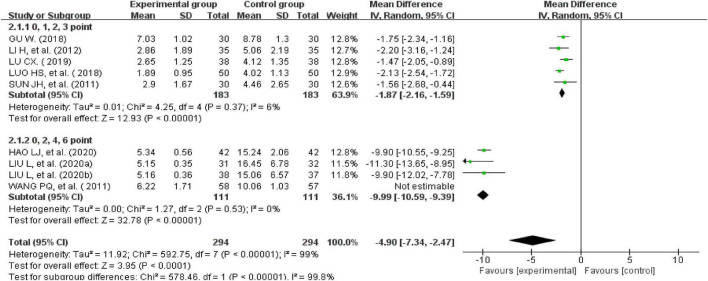
Forest plot of the total symptom score.

Nine studies recorded total symptom scores (24, 26–28, 35, 37, 38, 42, 43). There was significant heterogeneity across the studies when tested using I^2^statistics (*n* = 588, I^2^ > 50%, MD = -4.9, 95%CI [−7.34, −2.47], *Z* = 3.95, *P* < 0.00001). A subgroup analysis was performed according to the different weights given to the clinical symptom scores. The results showed that five studies assessed clinical symptom scores using 0, 1, 2, or 3 points (*n* = 366, I^2^ = 6%, MD = −1.87, 95% CI [−2.16, −1.59], *Z* = 12.93, *P* < 0.00001) (26–28, 42, 43). The other four studies evaluated clinical symptom scores using 0, 2, 4, or 6 points (*P* < 0.00001, I^2^ = 0%), indicating that the heterogeneity was still very high. However, the heterogeneity was significantly reduced after excluding one study (*n* = 222, I^2^ = 0%, MD = −9.99, 95% CI [−10.59, −9.39], *Z* = 32.78, *P* < 0.00001) (35). This means that the different weights given to the clinical symptom scores affected the results of the analysis. A subgroup analysis showed that the improvement in clinical symptom scores for IBS-D treated with external therapy using TCM alone was better than the improvement in clinical symptom scores for IBS-D in the control group (Figure 7).

#### Irritable bowel syndrome-severity scoring system score

**FIGURE 8:**
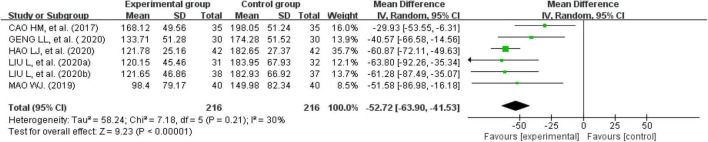
Forest plot of the score on irritable bowel syndrome-severity scoring system score questionnaire.

Eight studies reported IBS-SSS questionnaire scores (24, 25, 29, 30, 33, 37–39). One recorded them as the median and quartile; therefore, that study could not be used for this analysis (33). We were unable to consult with the original author to obtain further information. Finally, seven studies were analyzed. There was a significant heterogeneity across the studies when tested using I^2^ statistics (df = 5, *P* < 0.00001, I^2^ = 98%). The heterogeneity was significantly reduced after removing one study (*n* = 432, I^2^ = 30%, MD = -52.72, 95% CI [−63.9, −41.53], *Z* = 9.23, *P* < 0.00001) (29). The results showed that the improvement in the IBS-SSS questionnaire scores for external therapy of TCM alone was better than the improvement in the IBS-SSS questionnaire scores of the control group (Figure 8).

#### Irritable bowel syndrome-quality of life

**FIGURE 9:**
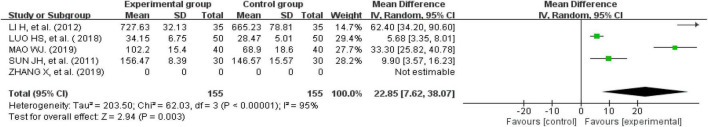
Forest plot of the score on Irritable bowel syndrome-quality of life scale.

Five studies recorded IBS-QOL questionnaire scores (25, 26, 28, 33, 43). One of these studies (33) reported IBS-QOL questionnaire scores as the median and quartile; therefore, that study could not be used for this analysis. The difference was statistically significant, because the experimental group had a positive effect on improving the IBS-QOL score (*P* < 0.05). Nevertheless, the heterogeneity was high (I^2^ > 50%) (Figure 9). A subgroup analysis based on intervention measures and duration showed decreased heterogeneity, but I^2^ was still > 50%.

### Adverse events

Among the 21 included studies, only six mentioned adverse events; among the six studies, three evaluated them as safe (26, 28, 43). One study recorded one case of anxiety and depression experienced by one patient in the experimental group (25). One study reported four cases of mild nausea and vomiting in the control group. The remaining studies reported seven cases of subcutaneous hemorrhage after acupuncture therapy. Correspondingly, two patients in the control group had a dry mouth, two had dizziness, and one had nausea (33). In particular, one patient developed severe diarrhea after using pinaverium bromide and was withdrawn from the study. In contrast, no adverse reactions were observed in the experimental group (31). All adverse reaction symptoms resolved spontaneously.

### Publication bias

**FIGURE 10:**
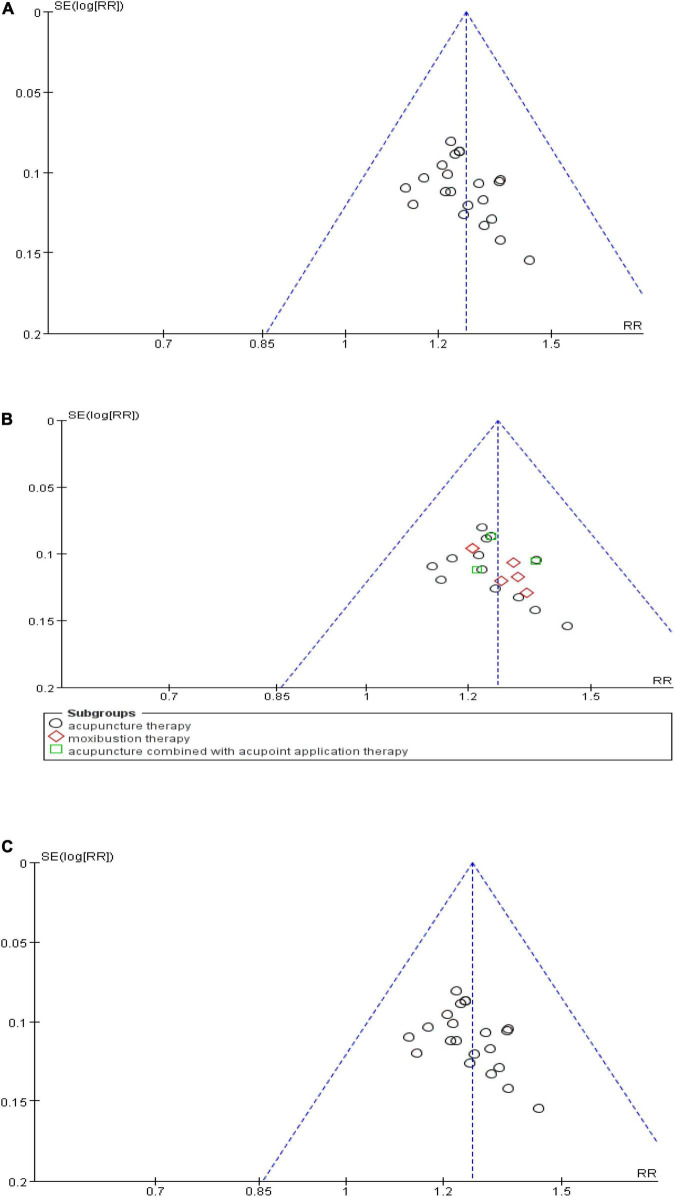
Funnel plot of publication bias. **(A)** Funnel plot of the total effective rate. **(B)** Funnel plot of the effective rate of subgroups. **(C)** Funnel plot of the clinical cure rate.

To detect a possible publication bias, we analyzed the funnel plot of more than 10 studies. The results showed that the morphological distribution on the left and right sides of the midline of the inverted funnel plot in the included studies was not symmetrical, indicating that the included studies had a potential publication bias (Figures 10A–C).

### Grading of recommendations, assessment, development, and evaluations certainty of evidence

The GRADE certainty of evidence for the primary and secondary outcomes is shown in Supplementary materials. The certainty of evidence was moderate for the total effectiveness rate and IBS-SSS score, low for the recurrence rate, and very low for the clinical cure rate, total symptom score, and IBS-QOL.

## Discussion

Traditional Chinese medicine, including Chinese herbal medicine and external treatment, can alleviate symptoms, improve cure rate, and reduce the recurrence of IBS-D (45). During this review, the positive effects of 21 external therapies involving TCM on trials involving patients with IBS-D were evaluated. The results showed that external therapy of TCM alone significantly improved the total effectiveness rate and clinical cure rate. Moreover, external therapy of TCM alone can reduce the recurrence rate of and improve both intestinal symptoms and QOL associated with IBS-D, with few side effects. Unlike previous research studies, we comprehensively summarized various external therapies of TCM. Furthermore, all the trials included in this study were high-quality clinical trials with a Jadad score of ≥3, which indicated a high quality of evidence.

Conventional Western medicine has a clear therapeutic mechanism and can temporarily improve the symptoms of diarrhea. However, the long-term effects remain unsatisfactory, and the recurrence rate is still quite high (40%) because of complex and unclear pathological mechanisms of IBS (46). External therapy involving TCM is a therapy that acts on the surface of the body or from outside the body to treat disease through corresponding somatic-visceral reactions. It is simple to use, results in few adverse reactions, and has a quick clinical efficacy; therefore, it is widely used to treat diarrhea. Furthermore, the World Health Organization recommends TCM for treatment of IBS (47). The external therapy of TCM during this study focused on acupuncture, moxibustion, and acupoint application; among these, acupuncture therapy and moxibustion therapy are the most commonly studied and mainly used clinical treatments for IBS-D. Based on the theory of somatic-visceral interactions, each external therapy with TCM has a similar efficacy when used to treat IBS-D. Additionally, several common clinical external treatment methods emphasize the role of stimulation of specific acupoints, and their mechanisms are also similar; therefore, they have similar effects on intestinal symptoms. Moreover, the sensitivity analysis showed that the total effectiveness and clinical cure rates of IBS-D treated with external therapy using TCM were relatively robust. It is noteworthy that the certainty of evidence was moderate for the total effectiveness rate and IBS-SSS. Similarly, the subgroup analysis revealed that the total clinical efficacy rates of acupuncture and moxibustion for treatment of IBS-D were better than that of Western medicine (*P* < 0.0001). Acupuncture is an important part of external treatment using TCM, which produces somatosensory stimulation at specific acupoints of the human body and releases the whole body to treat the intestinal tract. It induces multifaceted regulation to improve intestinal symptoms through complex mechanisms, such as inhibiting gastrointestinal motility, reducing visceral hypersensitivity, balancing the intestinal-brain axis, and regulating neurotransmitters and the immune system (48). The literature reports no need for acupuncture when the case is suitable for moxibustion. Moxibustion is another commonly used external therapy for patients with IBS-D. It uses the warm and medicinal power of ignited moxa to stimulate acupoints or specific parts of the surface of the body and promote the self-regulation function of the body. Moxibustion regulates intestinal inflammation, alleviates visceral hypersensitivity, and relieves visceral pain to improve functional gastrointestinal disorders (18, 49, 50). Acupoint application is a compound treatment method that integrates acupoints, meridians, and herbs that regulate meridians and improve blood circulation, thus exerting an effect on the intestinal system and improving diarrhea symptoms. Visceral hypersensitivity is the main pathogenesis of abdominal pain and diarrhea for patients with IBS-D; therefore, it has attracted increasing attention (51). Reducing visceral hypersensitivity to alleviate clinical symptoms is an important treatment strategy consistent with the mechanisms of acupuncture and moxibustion.

Unfortunately, the authors of the included studies did not explain the reasons for the lack of current clinical research data. IBS-D is a chronic, recurrent, and functional gastrointestinal disorder with no organic explanation. Its clinical trial is a complex process that involves clinical research and follow-up of participants, which involve high costs. Unfortunately, 20 to 30% of subjects withdrew from study participation (52). This is particularly true of studies with longer trial periods. Lack of long-term follow-up data was one limitation of this study. Another limitation of this study was that the random sequence allocation of the included studies was non-standard according to the summary risk of bias and publication bias graph, which may have caused selection bias. The funnel plot showed a skewed distribution, indicating a publication bias. Moreover, the accuracy of some results may have been affected by differences in the disease, reference standards for efficacy evaluation, and the unequal experience of TCM clinicians. Additionally, because of inevitable problems, such as database permissions, some gray bodies of literature were not retrieved.

External therapy of TCM for the treatment of IBS-D can alleviate abdominal symptoms, improve clinical effectiveness, and reduce recurrence with few side effects. Moreover, external therapy of TCM has a positive effect on improving QOL and can serve as an alternative treatment for IBS-D.

## Conclusion

The current evidence indicates that external therapy of TCM for IBS-D has positive efficacy and high safety. It is also simple, convenient, and low-cost. However, because of the limitations of the follow-up period and publication bias of the included trials, more rigorous clinical studies are necessary to further verify the long-term effects of external therapy of TCM.

## Data availability statement

The original contributions presented in this study are included in the article/Supplementary material, further inquiries can be directed to the corresponding authors.

## Author contributions

XW and XT contributed to the conception and design of the study. YoW and YuW searched the databases and extracted the data. XW, XL, and XM evaluated the studies for inclusion and data analysis. XW conducted the statistical analysis of the data and drafted the manuscript. YoW wrote sections of the manuscript. BZ and XT revised the manuscript. All authors read and approved the final version of the manuscript.

## Footnotes

1
https://www.crd.york.ac.uk/PROSPERO/
