# Supplementary material for: External therapy of traditional Chinese medicine for treating irritable bowel syndrome with diarrhea: A systematic review and meta-analysis
Source: Front Med (Lausanne). 2022 Aug 9;9:940328. doi: 10.3389/fmed.2022.940328 (PMC9396246; doi:10.3389/fmed.2022.940328)
Supplement: Supplementary file 1 [file Table_1.pdf]

Supplementary materials

Supplementary Table 1: Search strategies for each database

PubMed search strategy:

Search	Query
#1	(((((((((Irritable Bowel Syndrome[MeSH Terms]) OR (Irritable Bowel Syndromes[Title/Abstract])) OR (Syndrome, Irritable Bowel[Title/Abstract])) OR (Syndromes, Irritable Bowel[Title/Abstract])) OR (Colon, Irritable[Title/Abstract])) OR (Irritable Colon[Title/Abstract])) OR (Colitis, Mucous[Title/Abstract])) OR (Colitides, Mucous[Title/Abstract])) OR (Mucous Colitides[Title/Abstract])) OR (Mucous Colitis[Title/Abstract])
#2	diarrhea[Title/Abstract]
#3	#1 AND #2
#4	((((((((((external therapy[Title/Abstract]) OR (acupuncture[Title/Abstract])) OR (moxibustion[Title/Abstract])) OR (electroacupuncture[Title/Abstract])) OR (manipulation[Title/Abstract])) OR (Tui Na[Title/Abstract])) OR (massage[Title/Abstract])) OR (acupoint application[Title/Abstract])) OR (cupping[Title/Abstract])) OR (auricular point[Title/Abstract])) OR (hot compress[Title/Abstract])
#5	(((((randomized controlled trial[MeSH Terms]) OR (controlled clinical trial[Title/Abstract])) OR (random[Title/Abstract])) OR (randomly[Title/Abstract])) OR (randomized[Title/Abstract])) OR (control[Title/Abstract])
#6	#3 AND #4 AND #5

Embase search strategy:

Search	Query
#1	'irritable colon'/exp

#2	'irritable bowel syndrome':ab,ti OR 'irritable bowel syndromes':ab,ti OR 'syndrome, irritable bowel':ab,ti OR 'syndromes, irritable bowel':ab,ti OR 'colon, irritable':ab,ti OR 'irritable colon':ab,ti OR 'colitis, mucous':ab,ti OR 'colitides, mucous':ab,ti OR 'mucous colitides':ab,ti OR 'mucous colitis':ab,ti
#3	#1 OR #2
#4	diarrhea
#5	'external therapy':ab,ti OR 'acupuncture':ab,ti OR 'moxibustion':ab,ti OR 'electroacupuncture':ab,ti OR 'manipulation':ab,ti OR 'tui na':ab,ti OR 'massage':ab,ti OR 'acupoint application':ab,ti OR 'cupping':ab,ti OR 'auricular point':ab,ti OR 'hot compress':ab,ti
#6	'randomized controlled trial':ab,ti OR 'controlled clinical trial':ab,ti OR 'random':ab,ti OR 'randomly':ab,ti OR 'randomized':ab,ti OR 'control':ab,ti
#7	#3 AND #4 AND #5 AND #6

Cochrane Library search strategy:

Search	Query
#1	MeSH descriptor: [Irritable Bowel Syndrome] explode all trees
#2	(Irritable Bowel Syndromes):ab,ti,kw OR (Syndrome, Irritable Bowel):ab,ti,kw OR (Syndromes, Irritable Bowel):ab,ti,kw OR (Colon, Irritable):ab,ti,kw OR (Irritable Colon):ab,ti,kw OR (Colitis, Mucous):ab,ti,kw OR (Colitides, Mucous):ab,ti,kw OR (Mucous Colitides):ab,ti,kw OR (Mucous Colitis):ab,ti,kw
#3	#1 OR #2
#4	'diarrhea':ab,ti,kw
#5	#3 AND #4

#6	(external therapy):ab,ti,kw OR (acupuncture):ab,ti,kw OR (moxibustion):ab,ti,kw OR (Electroacupuncture):ab,ti,kw OR (manipulation):ab,ti,kw OR (Tui Na):ab,ti,kw OR (MASSAGE):ab,ti,kw OR (Acupoint application):ab,ti,kw OR (Cupping):ab,ti,kw OR (Auricular point):ab,ti,kw OR (HOT COMPRESS):ab,ti,kw
#7	#5 AND #6

Web of Science search strategy:

Search	Query
#1	(((((((((TS=(Irritable Bowel Syndrome)) OR TS=(Irritable Bowel Syndromes)) OR TS=(Syndrome, Irritable Bowel)) OR TS=(Syndromes, Irritable Bowel)) OR TS=(Colon, Irritable)) OR TS=(Irritable Colon)) OR TS=(Colitis, Mucous)) OR TS=(Colitides, Mucous)) OR TS=(Mucous Colitides)) OR TS=(Mucous Colitis)
#2	TS=(diarrhea)
#3	#1 AND #2
#4	((((((((((TS=(external therapy)) OR TS=(acupuncture)) OR TS=(moxibustion)) OR TS=(Electroacupuncture)) OR TS=(manipulation)) OR TS=(Tui Na)) OR TS=(massage)) OR TS=(Acupoint application)) OR TS=(Cupping)) OR TS=(Auricular point)) OR TS=(hot compress)
#5	(((((TS=(randomized controlled trial)) OR TS=(controlled clinical trial)) OR TS=(random)) OR TS=(randomly)) OR TS=(randomized)) OR TS=(control)
#6	#3 AND #4 AND #5

China National Knowledge Infrastructure (CNKI) search strategy:

Search	Query
#1	(SU='肠易激综合征'+'肠易激'+'腹泻型肠易激综合征'+'IBS'+'IBS-D') AND (SU='推拿'+'按摩'+'灸'+'针灸'+'针刺'+'电针'+'拔罐'+'耳穴'+'耳针'+'热敷'+'穴位'+'贴敷'+'脐疗'+'中医外治') AND (SU='随机对照试验'+'随机对照'+'随机'+'对照'+'临床试验')

Wan fang Database search strategy:

Search	Query
#1	主题: ('腹泻型肠易激综合征) and 主题: ('推拿' or '按摩' or '灸' or '针灸' or '针刺' or '电针' or '拔罐' or '耳穴' or '耳针' or '热敷' or '穴位' or '贴敷' or '脐疗' or '中医外治') and 主题: ('随机对照试验' or '随机对照' or '随机' or '对照' or '临床试验')

Chinese Biomedical Database (CBM) search strategy:

Search	Query
#1	"腹泻型肠易激综合征"[标题:智能]
#2	"推拿"[摘要:智能] OR "按摩"[摘要:智能] OR "灸"[摘要:智能] OR "针灸"[摘要:智能] OR "针刺"[摘要:智能] OR "电针"[摘要:智能] OR "拔罐"[摘要:智能] OR "耳穴"[摘要:智能] OR "耳针"[摘要:智能] OR "热敷"[摘要:智能] OR "穴位"[摘要:智能] OR "贴敷"[摘要:智能] OR "脐疗"[摘要:智能] OR "中医外治"[摘要:智能]
#3	"随机对照试验"[摘要:智能] OR "随机对照"[摘要:智能] OR "随机"[摘要:智能] OR "对照"[摘要:智能] OR "临床试验"[摘要:智能]
#4	#1 AND #2 AND #3

Chinese Scientific Journals Database (VIP) search strategy:

Search	Query
#1	(M=肠易激综合征 OR M=肠易激 OR M=腹泻型肠易激综合征 OR M=IBS OR M=IBS-D) AND (R=推拿 OR R=按摩 OR R=灸 OR R=针灸 OR R=针刺 OR R=电针 OR R=拔罐 OR R=耳穴 OR R=耳针 OR R=热敷 OR R=穴位 OR R=贴敷 OR R=脐疗 OR R=中医外治) AND (R=随机对照试验 OR R=随机对照 OR R=随机 OR R=对照 OR R=临床试验)

Supplementary Table 2. Modified Jadad scale

Items	Answer	Score
Was the method of randomization appropriate (table of random numbers, computer-generated, etc.)?	Adequate	2
	Unclear	1
	Inadequate	0
Was the method of blinding appropriate (identical placebo, active placebo, dummy, etc.)?	Adequate	2
	Unclear	1
	Inadequate	0
Was there a description of withdrawals and dropouts?	Yes	1
	No	0

Supplementary Table 3: Characteristics of excluded studies

**Study**	**Rationale for exclusion**
DAI L, et al. (2019)	The type of interventions (combined Western medicine) was ineligible for inclusion.
DENG PA, et al. (2018)	The type of interventions (not external therapy of TCM) was ineligible for inclusion.
GAO YM. (2012)	The type of interventions (combined Western medicine) was ineligible for inclusion.
JIN YQ, et al. (2017)	The sample size of study (< 60) was ineligible for inclusion.
KONG SP, et al. (2014)	The sample size of study (< 60) was ineligible for inclusion.
WU D, et al. (2021)	The type of comparators (external therapy of TCM) was ineligible for inclusion.
XU D, et al. (2015)	No results were reported and the study author could not be contacted to obtain records.
YANG M, et al. (2018)	The duration of treatments (12 days) was ineligible for inclusion.
YU LM, et al. (2020)	No outcome measures of interest were reported for meta-analysis.
YU LC. (2016)	No obvious and no described randomization.
ZHANG XX, et al. (2019)	The type of interventions (combined oral Chinese medicine) was ineligible for inclusion.
ZHENG HT. (2019)	The type of comparators (combined TCM) was ineligible for inclusion.
ZHONG F, et al. (2018)	No outcome measures of interest were reported for meta-analysis.
ZOU W, et al. (2019)	The duration of treatments (3 weeks) was ineligible for inclusion.
SHI H, et al. (2021)	No outcome measures of interest were reported for meta-analysis.
LI Y, et al. (2021)	The duration of treatments (2 weeks) was ineligible for inclusion.
CHEN Q, et al. (2021)	The method of randomization was inadequate (by visit order).
LI XL, et al. (2019)	No obvious and no described randomization.
SU Q, et al. (2019)	The method of randomization was inadequate (by visit order).
ZHANG XX, et al. (2019)	No obvious and no described randomization.
ZHANG ZJ, et al. (2018)	No obvious and no described randomization.
CHEN LL, et al. (2018)	No obvious and no described randomization.
SU DM, et al. (2018)	No randomization was reported or implied and the study author could not be contacted to obtain clarification.
XU L. (2018)	Dropouts were unstated and the study author could not be contacted to obtain clarification.
HAN J. (2017)	No obvious and no described randomization.
TAN ZY, et al. (2016)	The duration of treatments (2 weeks) was ineligible for inclusion.
SUN LJ, et al. (2016)	The method of randomization was inadequate (by visit order).
QIU WJ, et al. (2015)	No obvious and no described randomization.
LI XL, et al. (2015)	The study design among syndromes was not eligible for inclusion (not RCT).
LIU SY. (2014)	The method of randomization was inadequate (by visit order).

LI XL, et al. (2014)	No obvious and no described randomization.
LIU Y, et al. (2014)	No obvious and no described randomization.
LEI MN, et al. (2013)	The method of randomization was inadequate (by single number and double number).
DENG Q. (2012)	The method of randomization was inadequate (by visit order).
PEI LX, et al. (2012)	Dropouts were unstated and the study author could not be contacted to obtain clarification.
CHU HR, et al. (2011)	The duration of treatments (15 days) was ineligible for inclusion.
DONG J, et al. (2011)	The type of interventions (combined Western medicine) was ineligible for inclusion.
LIAN BL, et al. (2011)	The method of randomization was inadequate (by visit order).
ZHANG ZJ, et al. (2010)	No obvious and no described randomization.
ZENG YH, et al. (2010)	Dropouts were unstated and the study author could not be contacted to obtain clarification.
SHI XH, et al. (2010)	No obvious and no described randomization.
JIN GD. (2009)	No obvious and no described randomization.
ZHANG GZ, et al. (2004)	No obvious and no described randomization.
ZHAO CX, et al. (2020)	No obvious and no described randomization.
GUO GL, et al. (2010)	No obvious and no described randomization.
LI Y, et al. (2020)	No obvious and no described randomization.
HONG ZM, et al. (2011)	The method of randomization was inadequate (by single number and double number).
LIU YC. (2015)	No outcome measures of interest were reported for meta-analysis.

Supplemental Table 4. GRADE quality of evidence summary of the comparisons of external therapy of TCM versus Western medicine in IBS-D

Quality assessment							No of patients		Effect		Quality	Importance
No of studies	Design	Risk of bias	Inconsistency	Indirectness	Imprecision	Other considerations	External therapy of TCM	Control	Relative (95% CI)	Absolute		
**Total effective rate**												
21	randomised trials	serious	no serious inconsistency	no serious indirectness	no serious imprecision	none	877/983 (89.2%)	626/879 (71.2%)	RR 1.25 (1.20 to 1.31)	178 more per 1000 (from 142 more to 221 more)	⊕⊕⊕O MODERATE	CRITICAL
**Clinical cure rate**												
17	randomised trials	serious	no serious inconsistency	no serious indirectness	very serious	none	295/803 (36.7%)	148/699 (21.2%)	RR 1.66 (1.40 to 1.96)	140 more per 1000 (from 85 more to 203 more)	⊕OOO VERY LOW	CRITICAL
**Recurrence rate**												
5	randomised trials	serious	no serious inconsistency	no serious indirectness	serious	none	42/153 (27.5%)	69/107 (64.5%)	RR 0.44 (0.34 to 0.58)	361 fewer per 1000 (from 271 fewer to 426 fewer)	⊕⊕OO LOW	CRITICAL
**Total symptom score (Better indicated by lower values)**												
8	randomised trials	serious	very serious	no serious indirectness	no serious imprecision	none	294	294	-	MD 4.90 lower (7.34 to 2.47 lower)	⊕OOO VERY LOW	CRITICAL
**Total symptom score - 0, 1, 2, 3 point (Better indicated by lower values)**												
5	randomised trials	serious	no serious inconsistency	no serious indirectness	serious	none	183	183	-	MD 1.87 lower (2.16 to 1.59 lower)	⊕⊕OO LOW	CRITICAL

**Total symptom score - 0, 2, 4, 6 point (Better indicated by lower values)**												
3	randomised trials	serious	no serious inconsistency	no serious indirectness	serious	none	111	111	-	MD 9.99 lower (10.59 to 9.39 lower)	⊕⊕OO LOW	CRITICAL
**IBS-SSS (Better indicated by lower values)**												
6	randomised trials	serious	no serious inconsistency	no serious indirectness	no serious imprecision	none	216	216	-	MD 52.72 lower (63.90 to 41.53 lower)	⊕⊕⊕O MODERATE	CRITICAL
**IBS-QOL (Better indicated by lower values)**												
5	randomised trials	serious	very serious	no serious indirectness	serious	none	155	155	-	MD 22.85 higher (7.62 to 38.07 higher)	⊕OOO VERY LOW	CRITICAL
**Acupuncture therapy versus Western medicine**												
13	randomised trials	serious	no serious inconsistency	no serious indirectness	serious	none	566/643 (88%)	383/540 (70.9%)	RR 1.26 (1.18 to 1.34)	184 more per 1000 (from 128 more to 241 more)	⊕⊕OO LOW	CRITICAL
**Moxibustion therapy versus Western medicine**												
5	randomised trials	serious	no serious inconsistency	no serious indirectness	serious	none	182/200 (91%)	142/199 (71.4%)	RR 1.28 (1.16 to 1.41)	200 more per 1000 (from 114 more to 293 more)	⊕⊕OO LOW	CRITICAL
**Acupuncture combined with acupoint application therapy versus Western medicine**												
3	randomised trials	serious	no serious inconsistency	no serious indirectness	serious	none	129/140 (92.1%)	101/140 (72.1%)	RR 1.28 (1.14 to 1.43)	202 more per 1000 (from 101 more to 310 more)	⊕⊕OO LOW	CRITICAL
